# Ghrelin in Central Neurons

**DOI:** 10.2174/157015909787602779

**Published:** 2009-03

**Authors:** F Ferrini, C Salio, L Lossi, A Merighi

**Affiliations:** a Dipartimento di Morfofisiologia Veterinaria, Università di Torino, Via Leonardo da Vinci 44, 10095, Grugliasco; b Istituto Nazionale di Neuroscienze, Italy EU

**Keywords:** Ghrelin, GHSR, central nervous system, brain-gut hormone, feeding circuitry, memory, pain, neuroprotection

## Abstract

Ghrelin, an orexigenic peptide synthesized by endocrine cells of the gastric mucosa, is released in the bloodstream in response to a negative energetic status. Since discovery, the hypothalamus was identified as the main source of ghrelin in the CNS, and effects of the peptide have been mainly observed in this area of the brain. In recent years, an increasing number of studies have reported ghrelin synthesis and effects in specific populations of neurons also outside the hypothalamus. Thus, ghrelin activity has been described in midbrain, hindbrain, hippocampus, and spinal cord. The spectrum of functions and biological effects produced by the peptide on central neurons is remarkably wide and complex. It ranges from modulation of membrane excitability, to control of neurotransmitter release, neuronal gene expression, and neuronal survival and proliferation. There is not at present a general consensus concerning the source of ghrelin acting on central neurons. Whereas it is widely accepted that the hypothalamus represents the most important endogenous source of the hormone in CNS, the existence of extra-hypothalamic ghrelin-synthesizing neurons is still controversial. In addition, circulating ghrelin can theoretically be another natural ligand for central ghrelin receptors. This paper gives an overview on the distribution of ghrelin and its receptor across the CNS and critically analyses the data available so far as regarding the effects of ghrelin on central neurotransmission.

## INTRODUCTION

In the last years, ghrelin, a peptide ligand of the growth hormone secretagogue receptor (GHS-R), has gained increasing attention as a brain-gut hormone [82, 83, 86]. Ghrelin displays several biological effects such as regulation of feeding, gastric secretion and motility, fat mass deposition, and cell proliferation [4, 37, 42, 91, 160], but has gained increasing attention mainly for its effects on feeding behavior and metabolism [48, 54, 64, 121]. Whereas, it is widely accepted that the control of food intake occurs through activation of specific hypothalamic nuclei and the promotion of neuropeptide Y (NPY) and Agouti related protein (AgRP) expression [4, 5, 103, 142, 145, 148, 157], the distribution of GHS-R in central nervous system (CNS), and the modulation of neurotransmission in extra-hypothalamic areas suggest broader effects than originally predicted.

We will review below the most relevant data in the literature as regarding to the distribution and function of ghrelin and its receptor in central neurons linked to a neurotransmitter role of the peptide.

## HISTORICAL NOTES, BIOCHEMISTRY AND DISTRIBUTION OF GHRELIN IN CNS

Ghrelin is a 28-amino acid motilin-related peptide [4], originally purified from the rat stomach [81]. The peptide is characterized by the presence of an *n*-octanoylation on the hydroxy group of serine in position 3. This acylation is a post-transcriptional modification that is essential for binding to GHS-R [81, 145] to a point that ghrelin was originally supposed to be biologically active only in acylated form [81]. However, about 80-90% of circulating ghrelin is not acylated (des-acyl-ghrelin), and it still remains unclear whether or not des-acyl-ghrelin represents a precursor or a degradation product of the acylated peptide [65, 83]. Moreover, des-acyl-ghrelin does not replace radio labeled ghrelin at pituitary and hypothalamic binding sites, nor it seems capable of inducing growth hormone (GH) release. Therefore its biological role, if any, remains puzzling, and the possibility that des-acyl-grelin is a biologically active molecule acting through a specific, but yet uncharacterized receptor still remains a matter of debate. In support of this hypothesis, several *in vitro *studies have demonstrated that radio labeled ghrelin and des-acyl-ghrelin bind to the membranes of PC-3 prostate tumor cells, H9C2 cardiomyocytes and isolated adipocytes, none of which expressed the GHS-R [6, 24, 101]. In addition, ghrelin and des-acyl-ghrelin, at least in some cases, exhibit similar GHS-R independent biological activities, such as the inhibition of cell proliferation of breast carcinoma cell lines [23], the ionotropic effect on guinea pig papillary muscle [9], the promotion of bone marrow adipogenesis [138], the control of glucose output by primary hepatocytes [47].

Circulating ghrelin is mainly produced by X/A-like cells of the oxyntic stomach mucosa [3, 34, 41, 120, 154]. However, expression of the peptide has also been demonstrated in many other organs such as testis [136], ovary [19], placenta [55], kidney [100], pituitary [85], small intestine [34], pancreas [147], lymphocytes [61] and brain [33, 40, 90, 140].

In CNS, the main site of ghrelin synthesis (albeit at much lower levels than the stomach) is the hypothalamus. Expression of ghrelin in brain was initially established in the seminal paper by Kojima and co-workers [81]. Later, by using a combination of RIA and HPLC, Sato and co-workers clearly identified hypothalamic ghrelin [123]. By immunocytochemical techniques and colchicine pre-treatments, ghrelin expression was demonstrated in the internuclear space between the lateral hypothalamus, the arcuate nucleus (ARH), the ventromedial nucleus (VMN), the dorsomedial nucleus (DMN), the paraventricular nucleus (PVN) and the ependymal layer of the third ventricle [33, 66]. In these areas, ghrelin was localized in axon terminals innervating the ARH, VMN, PVN, DMN and the lateral hypothalamus. These axons made synapses with neurons expressing NPY/AgRP and pro-opiomelanocortin (POMC) [33]. However, according to other immunocytochemical studies, ghrelin is also synthesized by ARH neurons and these ghrelin-producing neurons display synaptic interactions with POMC, NPY and other ghrelin-containing nerve cells [57, 58, 63, 90]. These findings were confirmed by RT-PCR experiments [98] and, very recently, by the use of transgenic mice where the transcription regulatory regions of the ghrelin gene have been engineered to drive the expression of enhanced green fluorescent protein (EGFP) [74]. Finally, expression of the hormone in hypothalamus was also detected in human samples [95].

Outside the hypothalamus, ghrelin-immunopositive staining was observed in pyramidal neurons of layer V in the sensorimotor area and in the cingulate gyrus of the cerebral cortex, and the ghrelin mRNA was found in the sensorimotor cortex and in the dorsal vagal complex (DVC) of the medulla oblongata [66].

Localization in spinal cord and dorsal root ganglion (DRG) neurons remains to be ascertained with certainty, albeit after tyramide intensification we observed a limited number of positive medium-to-large DRG neurons and occasional cell bodies in laminae IV-IX of the spinal gray matter (unpublished data).

## BIOCHEMISTRY, FUNCTIONAL PROPERTIES AND DISTRIBUTION OF GHRELIN RECEPTOR(S) IN CNS

The GHS-R is a seven transmembrane-spanning domain G-protein coupled receptor that activates phospholipase C (PLC) *via* Gα_11_/G_q11_-protein [29, 67, 94, 113]. Consequently, PLC increases the intracellular Ca^2+^ levels, through inositol-3-phopshate (IP_3_)- and protein kinase C (PKC)-dependent pathways [2, 92]. Nifepidine and ω-agatoxin IIIA, but not conotoxin, inhibit the GHS-dependent Ca^2+^ increase, consistently with an activation of L-type calcium channels. This depolarizing effect is further strengthened by the inhibition of K^+^ channels, which probably involves the activation transient outward and delayed rectifier potassium channels [60].

In 1996, Howard and coll. [67] sequenced two cDNA clones encoding for two different GHS-R isoforms that were named type 1a and type 1b. Type 1a encodes for the full-length biologically active receptor. Conversely, type 1b encodes for a truncated isoform, lacking the transmembrane domains 6 and 7 of the type 1a, that is therefore thought to represent a non-functional receptor form [94]. In keeping with this assumption, binding affinity studies have shown that GHS secretagogues only bind GHS-R type 1a. Nonetheless, in HEK293 cells co-expressing full-length and truncated receptor isoforms it has been recently observed that GHS-R type 1b may play a regulatory/inhibitory role on type 1a receptor activity [26].

The distribution of GHS-R type 1a was investigated both in human and animal tissues using different techniques such as Western blotting, immunohistochemistry, *in situ* hybridization and radioimmunoassay [11, 49, 56, 97, 109, 126, 137, 164]. In these studies, GHS-receptors were localized in non-nervous organs/tissues (including adipose tissue, myocardium, adrenals, gonads, lung, liver, arteries, stomach, pancreas, thyroid, and kidney) as well as in CNS, with different levels of expression.

In CNS, GHS-R type 1a is highly expressed in the ARH and VMN of hypothalamus [11, 56, 66, 67, 97]. Co-expression of GHS-R with GH-releasing hormone, NPY, POMC, somatostatin, and tyrosine hydroxylase (TH) was also investigated in these areas [126, 137, 151]. Outside the hypothalamus, a positive receptor signal was observed in the cerebral cortex, dentate gyrus, CA2 and CA3 regions of the hippocampus, parafascicular thalamic region, substantia nigra, ventral tegmental area, raphe nuclei, nodose ganglion, and DVC [16, 17, 18, 40, 56, 66, 164]. By RT-PCR, Western blotting and immunohistochemistry we demonstrated the presence of GHS-R type 1a in mouse spinal cord [146]. After RT-PCR *in situ*, the mRNA was observed in neuronal cell bodies scattered across the base and neck of the dorsal horn. Following immunohistochemical labeling, positive neurons of corresponding sizes were observed in the same locations. Parallel patch clamp experiments demonstrated that these receptors were functional *in vitro*.

## GHRELIN AND FEEDING CIRCUITRY

According to a recent definition, a neurotransmitter is a molecule, released by neurons or glia that physiologically influences the electrochemical state of adjacent cells [129]. In this respect, the first evidence that ghrelin may act as neurotransmitter in CNS was provided by studies on the hypothalamic neurons involved in the control of feeding [72]. The physiological significance of the biological functions of ghrelin on nutritional homeostasis and metabolism has been authoritatively reviewed [32, 72, 108].

**Fig. (1):**
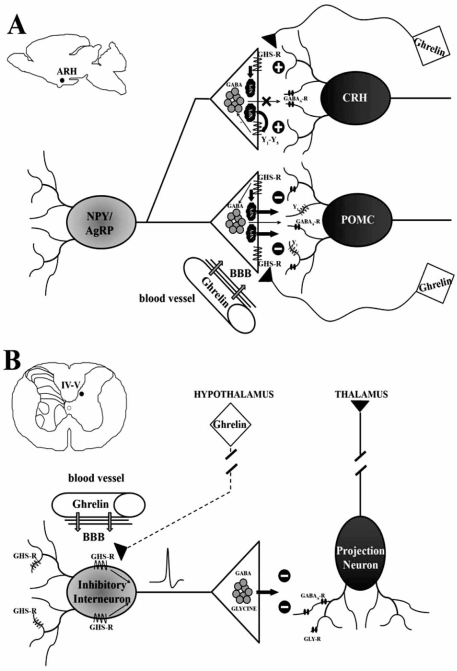
**Ghrelin modulation of inhibitory neurotransmission in central neurons.** A. In the hypothalamic ARH, GHS-Rs are expressed on axon terminals of NPY/AgRP-expressing inhibitory neurons. GHS-R activation at this level is supposed to induce the release of peptides such as NPY (thick arrows), more efficiently than aminoacids, such as GABA (thin arrow). NPY, in turn, inhibits anorexygenic POMC neurons by acting on postsynaptic Y1 receptors and disinhibits orexygenic CRH neurons by acting on pre-synaptic Y1-Y5 receptors, therefore reducing GABA release. The main source of ghrelin in this area of the brain is likely to be the circulating hormone that is capable to cross the BBB. However, a local source of ghrelin cannot be excluded, given that ghrelin-expressing neurons (squares) have also been described in ARH. B. In the spinal cord deep dorsal horn (laminae IV-V), GHS-Rs are mainly expressed at the somato-dendritic domain of local inhibitory interneurons. GHS-R activation leads to an action potential-dependent release of GABA and glycine onto putative projection neurons. The inhibition of these neurons reduces the outflow of sensory information (including pain) to higher centers (i.e. the thalamus). The source of spinal cord ghrelin is still under investigation, as it appears that ghrelin is locally expressed at very low levels (if any). Moreover, even though the existence of descending hypothalamic-spinal projections has been described, there are no data at present demonstrating a direct projection (dashed line) to the spinal cord from hypothalamic neurons synthesizing the peptide (square). Therefore also in spinal cord the most likely source of ghrelin remains the blood circulation.

Besides to these widely acknowledged functions, initial electrophysiological studies in hypothalamus strongly suggested that ghrelin also modulates neuronal excitability and synaptic transmission by acting on GHS-R type 1a [33, 117, 140, 144]. The main site of action of the hormone was found in ARH (Fig. **1A**), where ghrelin-positive terminals innervate a population of GHS-R type 1a expressing neurons [33, 125, 164]. Within ARH, ghrelin was shown to increase the firing rate of a population of neurons that were inhibited by the anorexigenic peptide leptin [117, 140]. The effect was partly blocked by the GHS-R type 1a antagonist (D-Lys3)-GHRP-6 [139]. The mechanism and the circuitry involved were elucidated in the interesting study of Cowley and coll. [33]. These authors used an acute slice preparation from hypothalamus obtained from two lines of transgenic mice in which NPY- or POMC-expressing neurons were genetically engineered to express a reporter fluorescent label. By this approach, ghrelin was shown to directly increase the firing rate of NPY/ AgRP neurons and to indirectly inhibit POMC neurons by facilitating the pre-synaptic release of γ-amino butyric acid (GABA) and NPY. Furthermore, in PVN, the main projection site of ARH neurons, ghrelin reduced the inhibitory tone of corticotropin-releasing hormone (CRH) neurons through activation of pre-synaptic NPY receptor Y1 and Y5, thus disinhibiting an important orexigenic pathway mediated by these neurons. Taken together, the above observations demonstrate that the effects of ghrelin on hypothalamic feeding circuitry are due to modulation of transmitter release from ARH neurons expressing NPY. Further confirmation was subsequently obtained after molecular genetic studies [28], where the effect of peripheral ghrelin on feeding behavior resulted to be reduced after NPY deletion (but not AgRP) and completely blocked in NPY/AgRP double knock-out animals. As observed by Cowley and Grove [32], the loss of ghrelin effect by simply switching off the NPY and AgRP genes excludes that the inhibitory effect of ghrelin onto POMC neurons is due to a direct facilitation of GABAergic transmission, but rather suggests that is largely mediated by peptide release.

Interestingly, by coupling patch-clamp recordings and single cell RT-PCR, van den Top and coll. [144] demonstrated that ghrelin induces regular bursts of action potentials with underlying oscillation of membrane potentials in NPY/ AgRP-expressing ARH neurons. This ghrelin-induced pacemaker activity is driven by low-threshold T-type Ca^2+^ channels and the bursting frequency is modulated by transient outwardly rectifying K^+^ currents. As demonstrated in the seminal paper of Poulain and Wakerley [114], burst activity is particularly effective in inducing peptide release from hypothalamic neurons. Therefore ghrelin should more efficiently modulate peptide release than amino acid release in ARH.

In a series of calcium imaging experiments on isolated ARH neurons, the activation of N-type Ca^2+^ channels *via* protein kinase A (PKA) was also proposed as a mechanism by which ghrelin may rise cytosolic Ca^2+^ levels in NPY expressing neurons [78]. Within the same population of neurons, ghrelin also elevates intracellular Ca^2+^ concentration by acting *via* phospholipase C and AMP-activated protein kinase [79, 80]. The increase of cytosolic Ca^2+^, besides changing membrane excitability, is generally known to activate many intracellular pathways that regulate protein synthesis and metabolism. In keeping with this notion, several lines of evidence suggest that ghrelin may regulate gene expression and *de novo* synthesis of other neurotransmitters [51, 103, 125]. In particular, intracerebroventricular (ICV) administration of orexigenic doses of ghrelin increases the mRNA level of AgRP and NPY in ARH neurons [103, 125], and a similar effect (even though conditional to the co-administration of corticosteroids) was observed in a hypothalamic organotypic culture model [51]. The stimulatory effects of ghrelin on NPY gene expression were abolished in the presence of cycloheximide, that blocks protein translation [51]. Furthermore in several hypothalmic nuclei involved in feeding control (ARH, PVN, DVM, VMN and lateral hypothalamic nuclei), ICV injections of ghrelin were shown to increase expression of Fos, a marker of neuronal activation [87]. Interestingly, the ghrelin-induced Fos expression was found in about 40% of NPY neurons in ARH [102, 148], but also in about 20% of orexin positive neurons in the lateral hypothalamic nucleus [139] and in a population of oxytocin positive neurons in PVN [107], suggesting that complex interactions occur between hunger-related neurotransmitters. It has been very recently shown that also the cannabinoid system is directly involved in the orexygenic effects of ghrelin in the hypothalamus. Indeed, blockade of the cannabinoid receptor type 1 prevents the ghrelin-mediated increase of hypothalamic AMP-activated protein kinase and inhibition of parvocellular PVN neurons [84]. Finally, it has been shown that ghrelin induces a rapid rewiring of ARH feeding circuits with a decrease of excitatory synaptic inputs to POMC neurons, while the inhibitory inputs were increased [111].

All together these studies suggest that the peptide acts as a neurotransmitter in the hypothalamus by modifying the output response of specific orexygenic neurons. Nonetheless, the biological effects of ghrelin in hypothalamus are not only restricted to modulation of neurotransmitter release at synapses, but also encompass a series of modifications in gene expression, cell metabolism, and synaptic connectivity. In addition, ghrelin was shown to indirectly influence the hypothalamic feeding circuitry by acting on the circumventricular organs. By using *in vitro* calcium imaging and patch clamp techniques, it was demonstrated that about 30% of the subfornical organ (SFO) neurons respond to ghrelin with an increase of cytosolic calcium concentration and spiking [115]. The depolarizing effect is dose dependent and seems to be triggered by the activation of a voltage independent non-selective cation conductance. SFO neurons project to the hypothalamus and are placed in a strategic site for interaction with blood circulating peptides, since SFO displays a leaky blood-brain barrier (BBB) [31]. Therefore SFO neurons may represent an important relay station between humoral signals originating in the gastrointestinal tract and ghrelin-responsive central neurons.

Broadly speaking, ghrelin-responsive hypothalamic neurons respond with an increase of food intake in the presence of a negative energetic status [103]. Even though maintenance of energy homeostasis is likely the most relevant effect in feeding behavior [13], ghrelin is also involved in the central regulation of other feeding-related aspects, such as the research of food for reward and the digestive function, by acting on midbrain and hindbrain areas, respectively [72, 108].

The effect of ghrelin in midbrain has been recently described in ventral tegmental area (VTA) and nucleus accumbens (Nacc) [1, 104]. Mesolimbic circuits located in VTA represent an important site for the generation of reward-seeking behaviors, including those to obtain reward from food. The main neurotransmitter underlying these behaviors, but also involved in the reward for sexual experience and drug assumption, is dopamine (DA) [12, 130]. DA is synthesized in VTA and substantia nigra and subsequently released onto Nacc and striatal neurons. Nanomolar concentrations of ghrelin were shown to increase the action potential frequency of VTA dopaminergic neurons *in vitro* when slices were obtained from wild type animals, but not *Ghsr^-/-^* mice [1]. In contrast with the experiments performed on ARH neurons [33, 111], ghrelin seems to act on VTA neurons independently from GABA-ergic neurotransmission, but rather inducing an increase and a significant plastic rearrangement of the excitatory input on DA neurons [1]. The results of these functional experiments were strengthened by histological studies in which co-expression of GHS-R type 1a and DA receptors in VTA, as well as in other dopaminergic brain areas (i.e. the hippocampus and substantia nigra), was demonstrated in a transgenic mouse model [71]. Interestingly, functional interactions between the two receptors amplify the DA signaling to VTA neurons. The ghrelin-induced potentiation of DA neurotransmission stimulates the overflow of DA in the Nacc, and this mechanism has been proposed to underlie the increased locomotor activity which is observed in the context of the feeding seeking behavior [68, 69]. Very recent evidence has been provided to show that the DA-enhancing effect is likely the result of interplay between ghrelin and the cholinergic system, but the underlying mechanisms still remain at least partly elusive [70].

The main site of ghrelin action in the hindbrain is the DVC, an autonomic centre that includes the nucleus tractus solitarius (NTS), the dorsal motor nucleus of the vagus nerve (DMV) and the area postrema (AP) [43]. NTS and AP receive visceral afferent inputs that drive a number of autonomic reflexes and relay visceral sensory information to other central stations that are involved in the control of energy balance [118, 124]. Moreover, DMV is a key centre for the parasympathetic control of the gastro-intestinal function and for central regulation of gastric secretion [119, 141]. In the DVC, ghrelin seems to affect both afferent and efferent signals. In keeping with this hypothesis, intravenously administered ghrelin activates the vagal afferent pathways and transection of the vagus nerve reduces the hyperphagic effects of peripheral ghrelin [37]. ICV administrations and intraparenchimal injections of the hormone in the DVC were shown to increase food intake [43]. GHS-Rs are expressed in DVC neurons [164], and the hyperphagic effect was proposed to be mediated by GHS-R expressing fibers running from NTS to the hypothalamus [38]. The signal activated by ghrelin reaches the ARH where it increases the release of noradrenaline [38]. On the other hand, ICV and intravenous injections of ghrelin induce an increase of Fos immunoreactivity in neurons of NTS, DMV and AP and stimulate gastric acid secretion and pancreatic secretion *via* the efferent vagal pathways [36, 88]. The existence of a subset of ghrelin-producing hypothalamic neurons that project to DVC has been revealed by immunohistochemistry, RT-PCR and tract tracing techniques [66]. Until recently there was a substantial lack of functional data in support to the idea that the hormone acts as a neurotransmitter within DVC. However, in keeping with this idea, Wang and coll. [149] have shown that ghrelin modulates cell excitability in DVC by using *in vivo* extracellular recordings.

## GHRELIN AND AROUSAL STATE

Several lines of evidence suggest that the effect of ghrelin in the hypothalamus is not only restricted to feeding behaviour and energy homeostasis, but also concerns the regulation of sleep-wake states [131]. Again, somehow contradictory results have been obtained by different authors. Systemic administration of ghrelin was shown to promote sleeping by increasing non-rapid eye movement sleep (NREMS) in mice [105], and slow wave sleep in humans [150]. Conversely, when administered intracerebroventricularly, ghrelin induced wakefulness and suppressed NREMS and rapid eye movements (REMS) in rats [132]. To identify the central site of ghrelin activity, Szentirmai and coll. [132] performed microinjections of the peptide in several hypothalamic areas implicated in sleeping and feed behavior. As predicted, injections in the lateral hypothalamus, medial preoptic area and PVN induced wakefulness and hyperphagia. To explain the apparent contradiction between pro-sleep and pro-awake effects two different alternative pathways can be hypothesized: the first one, controlled by circulating ghrelin, likely intervenes in growth hormone releasing hormone (GHRH) sleep-promoting mechanisms [105]; the second, controlled by central administered ghrelin, likely activates hypothalamic wake-promoting mechanisms with the intervention of orexin and NPY [106, 134]. Unfortunately, ghrelin knock-out mice were not very informative in clarifying the role of ghrelin in the arousal state, possibly as a consequence of the redundancy of the system [133]. In addition, very little is known concerning the molecular mechanisms and the underlying circuitry. Yi and coll. [158] have recently provided data in support to the notion that ghrelin modulates circadian activity by acting on the ARH-suprachiasmatic nucleus (SCN) axis. According to their experiments, peripheral administrations of the ghrelin synthetic analogue GHRP-6 inhibit the light-induced Fos expression in rat SCN, following activation of NPY inhibitory neurons in the ARH. Moreover, by measuring locomotor activity in mice, the same authors showed that GHRP-6 shortens the light-induced phase shift [158]. Since SCN is an important biological clock which is sensitive to external photonic, as well as non-photonic factors, the mechanism described above may be at the basis of ghrelin modulation of the arousal state. As to our knowledge, no data are available at present on the neurotransmitters and receptors involved. Nonetheless, the monoaminergic and cholinergic systems are likely to be involved based on the results of similar studies focused on the orexin mechanisms in sleep/wake state [106].

## GHRELIN AND MEMORY

Hippocampus, amygdala and dorsal raphe nucleus (DRN) are the main brain areas involved in learning and memory mechanisms. Initial evidence suggesting that ghrelin may have a role in mnestic functions was provided by the early work of Carlini and coll. [20, 21]. First, by using a behavioural test these authors showed that ICV injections of ghrelin increase memory retention [20]. To more precisely define the site of peptide action, the experiment was repeated after intraparenchimal injections of increasing concentrations of the hormone in hippocampus, amygdala and DRN [21]. A dose-dependent increase of memory retention was observed in each condition, with maximal effect in hippocampus. More recent data from the same laboratory suggest that the effects of ghrelin on memory could depend on the availability of serotonin (5-HT), since a 5-HT uptake inhibitor (fluoxetine) decreases both short and long term memory retention [22]. Underlying mechanisms have been partly elucidated by Diano and coll. [40], who showed that peripheral ghrelin injections rapidly rearrange synaptic organization with an increase in spine density in CA1 regions of hippocampus, similarly to hypothalamus [111] and midbrain [1]. Interestingly, comparable results were also obtained after analysis of spine density in wild type and ghrelin knock-out animals, giving further support to involvement of endogenous ghrelin in CA1 spine formation. Furthermore, the same authors showed that ghrelin promotes long term potentiation, a phenomenon that has a positive correlation with spatial memory and learning [40]. Animals that received ghrelin injections showed in fact enhanced performance in several behavioral memory tests that are dependent by hippocampus. Again, there is a lack of knowledge concerning the molecular mechanisms and transmitters involved. As discussed above, ghrelin receptors positively interact with DA and 5-HT receptors [22, 69] and D1/GHS type 1a co-expression has been reported in hippocampus [69]. Since the loss of cognitive functions in aging has been supposed to involve a decline in DA or 5-HT signaling [8, 15], ghrelin potentiation of these neurotransmitters in hippocampus may represent an interesting mechanism to intervene on memory impairment due to senescence or Alzheimer disease [8, 40]. On the other hand, the still limited number of data available so far leaves open the possibility that the effects of ghrelin on mnestic performances may be primarily related with feeding behavior. The model proposed by Diano and coll. [40] implies that circulating ghrelin is able to reach significant concentrations in the hippocampus. Furthermore, Carlini and coll. [20] have shown that injections of ghrelin in the hippocampus and DRN increased food intake in a dose-dependent manner. Altogether these data suggest that a gut-hippocampus axis may facilitate memory retention for the spatial localization of food [99].

## GHRELIN AND CENTRAL PAIN MECHANISMS

The relationship between ghrelin and pain has been the subject of very recent investigations, and knowledge in this field is still at its infancy. In general terms, currently available evidence indicates that ghrelin acts as an antinociceptive signal at both peripheral and central sites [59, 128, 146].

The early work of Sibilia and coll. [128] provided the first convincing evidences that ghrelin may has antinociceptive effects. By using the carrageenan model of acute inflammatory pain in rats, these authors showed that ICV injections of the hormone dose-dependently reduce mechanical hyperalgesia and paw oedema. A similar effect, albeit at slighter levels, was also observed after intraperitoneal, but not intraplantar injections. The action of ghrelin was reversed by ICV injections of the opioid antagonist naloxone. On these bases Sibilia and coll. [128] speculated about the possible mechanisms by which ghrelin centrally interacts with hypothalamic opioid-containing neurons. In particular, they postulated that peripherally administered ghrelin increases agrp synthesis and release [75], which, in turn, enhances release of β-endorphins from POMC neurons. Alternatively, or in addition to, they hypothesized that a ghrelin-dependent increase of nitric oxide (NO) synthase activity [46] may be responsible of the enhanced antinociceptive effects of endogenous opioids [62]. In our recent study [146], we provided evidence for the existence of central mechanisms of processing of nociceptive signals in spinal cord (Fig. **1B**). In mouse spinal cord slices, we have shown that ghrelin significantly enhances inhibitory (GABAergic/glycinergic) neurotransmission in a subpopulation of deep dorsal horn neurons, mainly localized in the medial aspect of laminae IV-VI. The effect is specifically due to interaction of ghrelin (in its biologically active octanoylated form) with GHS-R type 1a, since des-acyl-ghrelin was ineffective. We also showed that (D-Lys3)-GHRP-6 (a GHS-R type 1a antagonist) prevents the ghrelin effect. Interestingly, the antagonist reduces *per se* the frequency of inhibitory post synaptic currents (IPSCs), suggesting that a tonically active receptor is present in spinal cord. In addition, block of action potential-mediated neurotransmission with tetrodotoxin strongly reduced the ghrelin effect, indicating that in the dorsal horn, unlike the hypothalamus [33], ghrelin receptors do not exhibit an axonal distribution. Many deep dorsal horn neurons are wide dynamic range (WDR) neurons, and represent an important site of convergence for nociceptive and non-nociceptive stimuli [152]. Ghrelin-responsive neurons display the morphology of projection neurons that relay nociceptive information to supraspinal centers and represent the main output for lamina II interneurons [152]. It is also likely that at least some of them express the neurokinin 1 (NK1) receptor, the preferred substance P receptor [112]. It is thus conceivable that ghrelin, by increasing the inhibitory input onto lamina V projection neurons, leads to a reduction of sensory information outflow from spinal cord. To address this issue, we studied the effect of the peptide onto the capsaicin-induced increase of Fos immunoreactivity *in vitro* [146]. Consistently with the idea that ghrelin may exert anti-nociceptive central effects, capsaicin, a specific activator of nociceptive primary afferent fibers that induces Fos expression in dorsal horn [153], was unable to up-regulate Fos within the deep dorsal horn in the presence of ghrelin.

Still, considerable work is needed to depict a comprehensive picture of the role of ghrelin in pain central mechanisms. Nevertheless, the data reported above suggest that interaction with opioid/NPY expressing neurons should be further investigated to better understand the role of ghrelin as a pain modulator.

## GHRELIN AS A NEUROPROTECTIVE PEPTIDE

Several studies have shown that ghrelin has anti-apoptotic and protective effects on different types of cells subjected to ischemia/reperfusion injury. The hormone inhibits cell apoptosis in cardiomyocytes and endothelial cells [6, 10, 163], adipocytes [76], cells of the adrenal zona glomerulosa [93], osteoblastic MC3T3-E1 cells [77], pancreatic β cells [52, 53, 162], intestinal epithelial cells [110], and ovarian follicle cells [116]. Moreover, it exhibits protective effects against ischemia/reperfusion in gastric mucosa [14], pancreas [39], and the isolated rat heart [27, 44]. Recently, it has been observed that similar effects are also exerted in CNS, where ghrelin inhibits apoptosis during oxygen-glucose deprivation (OGD) [30]. Protection of hypothalamic neurons is achieved by inhibition of reactive oxygen species generation, stabilization of mitochondrial transmembrane potential, increase of the Bcl-2/Bax ratio, prevention of cytochrome c release, and inhibition of caspase 3 activation [30]. Similar effects were demonstrated in rat hippocampal neurons after ischemia/reperfusion injury, with increase of cell survival and reduction of death [89]. In keeping with the observations *in vivo*, primary cortical neurons are protected from apoptosis induced by lipopolysaccharide, glutamate, n-methyl-d-aspartate (NMDA) and H_2_O_2_. The anti-apoptotic effect is related to up-regulation of Bcl-2 and heat-shock protein 70 (HSP70), and inhibition of caspases 8, 9, and 3 upon binding to GHS-R type 1a [96]. Similarly, apoptosis is blocked in rat pheochromocytoma (PC12) cells, where ghrelin reduces apoptosis by inducing the expression of HSP70 that, in turn, inhibits signal-regulating kinase 1 (ASK1) activity and ASK1-mediated caspase 3 activation [156].

The effect of ghrelin on neuronal survival is not limited to neuroprotection, but it also extends to cell proliferation in both the embryonic and adult nervous system [73, 122, 159, 160, 161]. Both the acylated and the des-acylated form of the peptide have been shown to promote embryonic spinal cord development and neurogenesis [122]. In the adult rat nervous system, ghrelin stimulates *in vitro* and *in vivo* neurogenesis in DMN and NST, after cervical vagotomy [160, 161]. Finally, the synthetic GHS-R agonist hexarelin and ghrelin itself stimulate the incorporation of ^(3)^H-thymidine in adult rat hippocampal progenitor cells, as an index of increased cell proliferation. In addition, hexarelin, but not ghrelin, also shows a significant inhibition of apoptosis and necrosis [73]. The effects of ghrelin on cell proliferation appear to be linked not only to GHS-R type 1a activation, but also to other yet uncharacterized peptides [73, 122]. All together, these data indicate that ghrelin may also act as a survival factor that promotes neurogenesis, preserves tissue integrity from ischemic injuries, and inhibits apoptosis.

## GHRELIN PASSAGE ACROSS THE BBB

The effects of ghrelin on synaptic transmission, neuronal excitability and intracellular calcium levels suggest a transmitter-like behavior. However, as mentioned above, a determinant factor for defining a molecule as neurotransmitter is the existence of an endogenous source in neurons or glia. The question whether or not a central source of ghrelin does indeed exist and plays significant function in neurotransmission is still under debate. In a previous paragraph, we reviewed the most relevant data on expression of the peptide in CNS as established after different experimental approaches. Unfortunately, these data are often conflicting among each other, and a general consensus concerning the anatomical distribution of ghrelin-containing neurons and their projections is still far to be achieved. The reasons for these discrepancies should not only be sought in different sensitivities among the experimental procedures employed for localization, but, more importantly, in the low level of ghrelin expression in CNS [81]. In addition, several studies on the central effects of ghrelin concerned a number of brain areas where local synthesis has not yet convincingly been demonstrated. This is the case of many extra-hypothalamic areas, including hippocampus, midbrain, hindbrain and spinal cord. Therefore, in these areas circulating ghrelin is likely to be the only endogenous source of the peptide. In this context, the possibility that circulating ghrelin may cross the BBB has been the obvious subject of several investigations [86, 108]. Apart from the SFO and related circumventricular regions where the BBB is incomplete [45], it is currently accepted that peptide molecules are not capable to cross the BBB. However, intravenous ghrelin injections have been reported to induce an increased Fos expression also in non-circumventricular areas [135]. To explain the passage of ghrelin across the BBB, the existence of specific transporters has been proposed by Banks and coll. [7]. These authors studied in mice the blood-to-brain and the brain-to-blood passage of intravenously and ICV injected acylated and desacylated radioactive-labeled ghrelin peptides. From these studies, it appeared that octanoylated mouse ghrelin easily crosses the barrier but only in the brain-to-blood direction by a saturable carrier system, whereas des-acyl-ghrelin displays an opposite behavior due to a non-saturable mechanism. Interestingly in mice, human ghrelin, which differs from the mouse peptide for two amino acid residues, can be bidirectionally transported across the BBB by a saturable system [7, 40]. Therefore it appears that the transport of acylated and des-acylated ghrelin across the BBB is finely regulated by complex mechanisms, involving both active and passive transport. This, in turn, suggests that the bidirectional transport of ghrelin peptides from brain to extra-nervous compartments and *vice versa* is strictly linked to the array of biological functions that the peptide potentially exerts both at centre and periphery. However, whereas the physiological significance of the passage of the octanoyl ghrelin from the hypothalamus to the general circulation may be convincingly explained by taking into consideration the hormone-like functions of the peptide, retention of des-acyl-ghrelin in the brain seems less obvious. An interesting possibility is that circulating des-acyl-ghrelin, once entered the brain, is sequestered by specific cells that convert the peptide into the active octanoylated form. Octanoylation is a unique process in animal cells, which requires an appropriate biochemical machinery to be brought to completion. The key enzyme in the process has remained elusive, and this has rendered particularly difficult the identification of the cells, if any, that may be involved in the conversion process. Two very recent reports on the characterization of GOAT, the ghrelin O-acyl transferase, as a conserved orphan membrane-bound O-acyl transferase that specifically octanoylates serine-3 of the ghrelin peptide, open a new avenue in the study of the biological effects of the hormone [50, 155].

Descending fibers from the hypothalamus are most likely the main source of ghrelin in spinal cord, as it appears to be the case for certain orexygenic peptides, such as hypocretin 1 and 2 (or orexin A and B) [35, 143]. This would be in accordance with studies demonstrating the existence of direct descending projections connecting the hypothalamus and the spinal cord dorsal horn [25]. However, even though more specific studies are needed, our immunocytochemical studies do not completely rule out the possibility that the peptide is also expressed in DRGs and/or intrinsic neurons of spinal cord (unpublished data).

In summary, according to our current knowledge, circulating ghrelin is the most important source of the peptide in brain and spinal cord. Since circulating ghrelin is mostly represented by des-acyl-ghrelin, acylation mechanisms in CNS should be the subject of further studies. Interestingly, transcripts for GOAT occur predominantly in stomach and pancreas [50], therefore suggesting that CNS is unlikely to be a major site of ghrelin acylation.

## CONCLUSIONS AND FURTHER DIRECTIONS

Given that ghrelin synthesized in the stomach and released into the general circulation seems to be the main source of the peptide acting on central neurons, one has to keep clear in mind that most of the effects of ghrelin in brain are likely due to a gut peptide released in the bloodstream under fasting conditions [83]. Therefore the central effects of the peptide should be placed within a more general framework of adaptive mechanisms facilitating feeding behavior. The role of ghrelin in central neurons has encountered increasing interest in recent years, and a coherent scheme of ghrelin functions is taking shape. It is clear that the latter cannot be confined to the GH-releasing effects or the increase of food intake. The arrays of effects produced by ghrelin seem to converge in concert to put the organism in the condition of recovering from a negative energetic status. Nonetheless, the neuropharmacology of this peptide is still poorly understood, and much more work should be done in this direction. In particular, it will be of interest to establish whether or not des-acyl ghrelin is indeed a neuroactive molecule, if receptors subtypes other than GHS-R type 1a are responsible for ghrelin and/or des-acyl-ghrelin central effects, and how can acylation and des-acylation be regulated in the brain.

Heterogeneity of ghrelin receptors has already been documented outside the CNS [102]. If similar data will be obtained also in CNS, they may add new insights in the complex pattern of ghrelin functions in the brain.

## ABBREVIATIONS

5-HT: = Serotonin
AgRP: = Agouti related protein
AP: = Area postrema
ARH: = Arcuate nucleus
ASK1: = Signal-regulating kinase 1
BBB: = Blood brain barrier
CNS: = Central nervous system
CRH: = Corticotropin-releasing hormone
DA: = Dopamine
DMN: = Dorsomedial nucleus
DMV: = Dorsal motor nucleus of the vagus nerve
DRG: = Dorsal root ganglion
DRN: = Dorsal raphe nucleus
DVC: = Dorsal vagal complex
EGFP: = Enhanced green fluorescent protein
GABA: = γ-amino butyric acid
GABAA-R: = GABAA receptor
GH: = Growth hormone
GHRH: = Growth hormone releasing hormone
GHS-R: = Growth hormone secretagogue receptor
GLY-R: = Glycine receptor
GOAT: = Ghrelin O-acyl transferase
HSP70: = Heat-shock protein 70
ICV: = Intracerebroventricular
IP_3_: = Inositol-3-phopshate
Nacc: = Nucleus accumbens
NK1: = Neurokinin 1
NMDA: = n-methyl-d-aspartate
NO: = Nitric oxide
NPY: = Neuropeptide Y
NREMS: = Non-rapid eye movement sleep
NTS: = Nucleus tractus solitarius
OGD: = Oxygen-glucose deprivation
PKA: = Protein kinase A
PKC: = Protein kinase C
PLC: = Phospholipase C
POMC: = Pro-opiomelanocortin
PVN: = Paraventricular nucleus
REMS: = Rapid eye movement sleep
SCN: = Suprachiasmatic nucleus
SFO: = Subfornical organ
TH: = Tyrosine hydroxylase
VMN: = Ventromedial nucleus
VTA: = Ventral tegmental area
WDR: = Wide dynamic range
